# The Week's Work

**Published:** 1910-04-23

**Authors:** 


### The Week's Work.

### THE HOSPITALS' REFERENCE ANNUAL.

This week the 1910 edition of *Burdett's Hospitals and Charities* is issued, and in the preface to the new edition the editor draws attention to one or two points which are worth the serious attention of hospital workers at the present time. The issue of the Report of the Poor Law Commissioners, and especially the publication of the Minority Report, together with the active propaganda which the supporters of the Webb proposals are carrying on, make it essential that the authorities of all hospitals and agencies for the relief of the sick should join hands and evolve a plan of co-operation by which the work of the Commissioners, and the legislation that must admittedly follow in the near future, will be rendered really useful and beneficial to the public. Such scheme of co-operation, as Sir Henry Burdett has already pointed out in his address before the Home Hospitals Association, must ensure one perfected, complete, and adequate system of medical relief, calculated to support and strengthen, not break down and annihilate, the Voluntary hospital system. No one who is interested in this absorbing problem should fail to read the introductory articles which preface the reference chapter and statistical tables in the edition of 1910. From the information therein given, and from a study of the financial tables appended, the position of the voluntary hospitals in this country is easily ascertainable, and it is at once apparent to anyone who follows the figures closely, and especially to anyone who compares the situation in this country with that in America, as illustrated in Dr. Goldwater's interesting article on " State-Aided Hospitals in the United States and Canada," that the Voluntary system has served excellently, so far as supporting the hospitals goes, during the last twelve years. In the second chapter the enormous awakening influence of the great funds and the League of Mercy is clearly shown, and it is well pointed out that the keener public interest in hospital questions becomes, and, above all, the closer the co-operation between hospital workers themselves, the better becomes the position of the various institutions both from a point of view of general efficiency and financial support. This chapter is a closely reasoned argument in support of the Voluntary system, which should be carefully digested by anyone who ventures to criticise a principle which has been proved to be financially sound since it guarantees to two thousand charities a collective annual income of twelve million sterling, with an average excess of income over expenditure of more than a million sterling per year.

### SOME ADDITIONS AND THEIR MEANING.

We do not intend to give, in these paragraphs, a review of the edition of 1910: we content ourselves with a few notes to draw attention to some of the outstanding features of the work. At the present moment it is more imperative than ever before that those who are interested in what may be called " Charity Problems of the Day " should possess a reference manual which is exhaustive and, above all, absolutely reliable. That want is supplied by Burdett's *Hospitals and Charities*, and it is only inevitable, as the years pass, that the work of compiling and editing it should become more laborious and demand more self-sacrifice and care on the part of all those concerned in its production. In the present edition many new features are to be noticed, all of which, we feel sure, will be welcomed by those who are honestly interested in hospital questions. One section of these new additions deals with orphanages, homes, and refugees, a branch of the field of charity in the management of which more economical and up-to-date administration might with advantage be introduced. The article on hospital construction during 1909 sketches the main features of advance in hospital design and building which have marked the construction programme of the past year not only in this country, but elsewhere, while another chapter deals very fully with Hospital Sunday. The section on Poor Law infirmaries, specially topical at the present moment, is much more complete and full than in former editions, while the same may be said of the excellent article on nursing. Since its first publication *Hospitals and Charities* has been issued at a price below the cost of publication, and it has now been decided to raise the price to half-a-guinea. No one who has looked through the present year's edition and marked the increasing usefulness of the work, not merely as a manual of reference but as an authoritative work on the hospitals of the day and the many problems that loom for solution in the hospital world at the present time, will consider that price too extravagant.

### THE NATIONAL SANATORIUM ASSOCIATION.

The fourth annual report of the National Sanatorium Association, founded in 1905 for the establishment and maintenance of workers suffering from phthisis, deals almost wholly with the work at the one sanatorium controlled by the Association so far, namely, the institution at Benenden, in Kent. The year has been mainly devoted to detail work, and various much-needed improvements, structural and otherwise, have been completed at the sanatorium; but the accommodation is still far from effective, and the Committee earnestly appeals for subscriptions towards the building fund. The Association has arranged a mortgage for £8,000 at 4 per cent. with the United Kingdom Postal and Telegraph Service Benevolent Fund, and with this loan all previous mortgages were paid off and outstanding liabilities cleared, so that the financial position of the Association is at the present time much more satisfactory. A fair amount of active propaganda work has been done during the past year, and we are glad to notice that all the popular lectures given under the auspices of the Association have been well attended. It is by such means, by active propaganda work, that the Association will do the most good, both to itself and to the important subject of the prevention of consumption. It is worth while once more to point out the conditions under which beds are maintained at Benenden. Beds are allotted to Workers' Associations—that is, any combination of workers, temporary or permanent, among whom the principle of mutuality operates, and which provides that the patient's maintenance is paid for by his fellows—in order of application, at an annual charge of £65 per bed, or, temporarily, at a charge of 25s. per week. Organisations which have maintained a bed for a year or more can obtain one or more temporary beds at the same weekly rental. Increased accommodation will be available almost immediately, and the Committee appeals to all friendly societies to assist in maintaining the institution by acquiring beds. The results obtained at the Benenden Sanatorium are fully equal to those at other institutions of a similar character, and the treatment of patients is in every way satisfactory. A great deal of attention is paid to exercise and work on the part of the patients, and during the year many structural improvements have been effected entirely by means of patients' work. The sanatorium and the Association are worthy of the help that may be given them by anyone interested in the work of fighting consumption in this country.

### THE COST OF OPERATING GLOVES.

The price of rubber has, as everyone knows, advanced by leaps and bounds during the past few months. This fact is brought nearly home to those in surgical practice, and especially to hospital committees, by the steady rise in the cost of rubber operating gloves. These market fluctuations will cost the hospitals of the country many hundreds of pounds; the greater part of this will go as extra profit into the pockets of the manufacturers or the middlemen, who are using the rubber boom as an excuse for an extortionate increase in the price of the gloves. Pairs costing in the usual way three shillings and sixpence each have been advanced in price a shilling, fifteen, and even eighteen pence by many firms. At first sight this looks not unreasonable, for the rise is but 30 or 40 per cent., and the cost of raw rubber has risen a good deal more than that. But the actual quantity of rubber in a pair of fine operating gloves is very small indeed, and the greater part of the cost of making them probably lies in the fashioning and finish of the goods. This item, it is to be presumed, has not been affected by the recent excitement in Mincing Lane. Beyond that it is to be remembered that the cost price to the manufacturer and the wholesale price at which he sells them to the distributing agencies and the retailers are much below the price that is paid by the consumer. Yet the rise in price to the latter is all sheer profit to somebody. Those hospitals and surgeons that have laid in a stock sufficient to last some time may be congratulated on their forethought.

### L'INFIRMIERE.

Belgian hospitals are to be congratulated upon the institution of a new hospital and nursing organ, *L'Infirmière*, which is described as the organ "des écoles belges d'infirmirie laïque," and is published monthly at an annual subscription of 2 francs. It is a popular journal, and the first issue is a modest but interesting number. The offices of the administration are 206 Chaussée de Gand, Brussels, and the editorial committee includes such prominent institutional workers in Belgium as Drs. Carlier, Dejardin, and van der Velde and Miss Cavell. Dealing with the programme of the paper, the editors point out that Belgium has progressed rapidly during the last decade in hospital construction and management. Above all, the nursing arrangements have been brilliantly reformed, and nursing as a profession has come into its own. In course of time the infirmary servants, who correspond largely to our scrubbers and ward maids, and who have hitherto as largely acted as nurses in the older Belgian hospitals, will be replaced by properly trained nurses. The object of *L'Infirmière* will be to encourage and develop the movement which strives to ameliorate the conditions under which nursing is carried on and to create a wider interest in the doings of the profession of nursing and in everything connected with institutional work than at present exists. We cordially congratulate the editorial committee upon the excellence of its first number, with parts of which we hope to deal in a future issue, and we wish them every success in the future.

### THE DRUG MARKET.

Business has been somewhat quieter this week, and there have been very few price changes of importance. The price of cod-liver oil is still maintained, but there is not a good demand; up to the present the catch of codfish in the Norwegian fishing grounds has not been very much smaller than last season, but the yield of cod-liver oil has been about 10,000 barrels less than last season, so that prices may not recede to any considerable extent from their present level. Saffron is again rather dearer, owing to the effect on the bulbs of the unfavourable weather which has prevailed in Spain. Morphine is, if anything, the turn dearer, and the price of opium is well maintained. Linseed and linseed oil are dearer. Although there is not much demand at the moment for cascara sagrada, there are indications that values may advance before long. Next week it should be possible to say something definite about the spirit duties, and consequently about the price of rectified spirit and spirituous preparations.

